# P-1846. Differences in Type 6 Secretion System Composition among Serogroups of Neonatal Meningitis *Escherichia coli*

**DOI:** 10.1093/ofid/ofae631.2007

**Published:** 2025-01-29

**Authors:** Julia Ienes-Lima, Sonsiray Álvarez-Narváez, Nicolle Barbieri, Lisa Nolan, Catherine Logue

**Affiliations:** University of Georgia, Athens, Georgia; University of Georgia, Athens, Georgia; University of Georgia, Athens, Georgia; University of Georgia, Athens, Georgia; University of Georgia, Athens, Georgia

## Abstract

**Background:**

Neonatal meningitis is a public health challenge for pediatricians. Neonatal meningitis *Escherichia coli* (NMEC) is the most common Gram-negative bacterial cause of sepsis and meningitis in newborns in the United States. NMEC strains can replicate in macrophages, survive in the bloodstream and traverse the blood-brain barrier, resulting in meningitis. Most NMEC cases are associated with serogroups O18, O83, O7, O12, O1, and O45 (Logue et al., 2012). Two clusters of the Type 6 Secretion System (T6SS-I and T6SS-II) were identified in an NMEC O18:K1 strain, and studies have demonstrated that T6SS plays an essential role in the pathogenesis of several bacterial species. However, there is still a lack of knowledge regarding the function of some T6SS genes. Here, we investigated the role of T6SS in different NMEC serogroups to identify specific genes that may be used as a potential target for an efficacious prevention strategy.

Characteristics of NMEC strains

**Figure 1.**
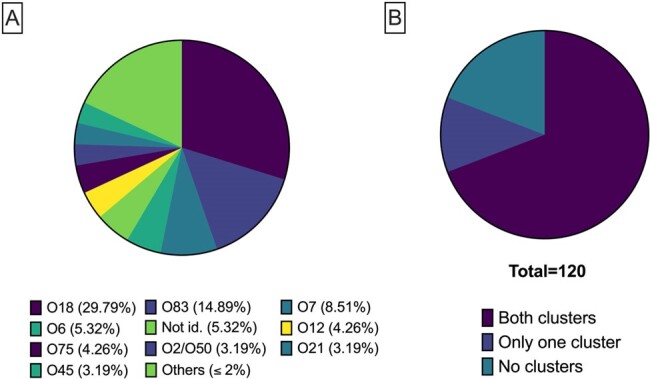
Characteristics of NMEC strains used in this study. A) Percentage of serogroups; B) Prevalence of T6SS among the NMEC isolates.

**Methods:**

A total of 120 NMEC genomes were used in this study (n=78 from the Logue Lab collection; n=42 publicly databases). The T6SS clusters were identified through BLAST analysis using NMEC RS218 and O18 as reference genomes. To establish the presence of a specific cluster, a query cover and percentage identity threshold of 60% and 70% were set. The sequence comparisons were performed using the Artemis Comparison Tool (ACT).

**Results:**

The serogroup O18 was the most abundant (23%; n=28), followed by O1 (22%; n=26), and O83 (12%; n=14). Analysis examined the prevalence of both T6SS clusters in the NMEC genomes and identified that they were present in 69% (n=83) of the genomes, while 12% (n=14) have only one of the clusters, and 19% (n=23) have no clusters. The presence of both T6SS clusters was observed in the serogroups O18, O14, and O2/O50. In contrast, no gene of any cluster was identified in serogroup O7. To understand the role of variation among the serogroups, *in vitro* analyses were performed. The results indicate differences in survival within THP-1 macrophages-like among distinct serogroups.

**Conclusion:**

Our results support the hypothesis that T6SS components differ among the NMEC serogroups, potentially impacting their pathogenicity and ability to cause meningitis. These findings have significant implications for development of a prevention strategy for neonatal meningitis.

**Disclosures:**

**All Authors**: No reported disclosures

